# The Influence of Pore Characteristics on the Mechanical Properties of 3D-Printed Concrete Based on the Phase-Field Method

**DOI:** 10.3390/ma19122637

**Published:** 2026-06-18

**Authors:** Lei Luo, Yao Li, Wenbin Xu, Yuchi You, Wenqiang Xu, Deyong Hu

**Affiliations:** 1 Hunan Provincial Water Resource Development & Investment Co., Ltd., Changsha 410128, China; ll1246599@163.com; 2 College of Water Resources and Civil Engineering, Hunan Agricultural University, Changsha 410128, Chinaxwqiang@hhu.edu.cn (W.X.); 3 College of Water Conservancy and Hydropower Engineering, Hohai University, Nanjing 210098, China

**Keywords:** 3D-printed concrete, phase-field method, pore characteristics, interlayer properties, crack evolution

## Abstract

The interlayer pores of 3D-printed concrete (3DPC) significantly weaken its macroscopic mechanical properties. In this study, the phase-field cohesive zone model (PF-CZM) is employed as a numerical tool to systematically investigate the weakening mechanisms and crack evolution behavior associated with pore characteristics, including pore size, morphology, spatial orientation, and arrangement, through single-factor numerical simulations with different pore numbers. The results demonstrate that the degradation induced by a single pore is controlled by its effective projection length in the direction perpendicular to the principal tensile stress, with horizontal flat pores being the most detrimental under the same porosity. In the multi-pore system, the connection angle between pores, rather than their spacing, is the key factor determining structural degradation, and a horizontal collinear arrangement is prone to triggering brittle fracture. Furthermore, locally aggregated small pores can form combined defects, whose strength-weakening effect surpasses that of isolated large pores, thereby triggering crack path competition and leading to asymmetrical structural failure. This study reveals the fracture mechanisms driven by complex pore configurations and provides a reference for strength prediction of 3DPC.

## 1. Introduction

3D-printed concrete (3DPC) has attracted considerable attention owing to its advantages in formwork-free construction, high geometric customization capability, and improved material utilization efficiency [1,2,3]. However, the layer-by-layer extrusion and deposition process inevitably generates distinct interlayer interfaces between adjacent printed filaments, accompanied by air entrainment, insufficient interfacial overlap, and inadequate material compaction. These processes give rise to defects such as pores and microcracks in the interfacial regions. Such interlayer defects weaken interfacial bonding, reduce the effective load-bearing area, and induce pronounced mechanical anisotropy, thereby becoming a critical factor limiting the structural application of 3DPC [4,5,6].

In recent years, significant progress has been made in characterizing the interfacial pore features of 3DPC. With the aid of non-destructive three-dimensional imaging techniques, such as X-ray computed tomography (X-CT) [7], researchers have found that the pore network within 3DPC exhibits a high degree of heterogeneity [8]. Unlike the nearly spherical pores commonly observed in conventional cast-in-place concrete, the mesoscopic pores in 3DPC generally present flattened ellipsoidal or elongated spheroidal morphologies due to extrusion forming, interlayer deposition, and compression from the upper deposited layers. Their equivalent diameters are mainly distributed within the range of 0.06–1.2 mm [9]. Previous studies have shown that pores with diameters greater than 0.1 mm are more likely to be elongated and preferentially oriented along the printing direction [10], while pores larger than 1.0 mm generally exhibit a compactness lower than 0.2 [11], and the average aspect ratio of interlayer pores can exceed 3. In addition, pore orientation also exhibits a clear statistical bias; for example, pores whose major axes form angles of 60–90° with the vertical printing direction can account for up to 58% [9]. When the pore size further increases, such as beyond 0.16 mm, pores in the interlayer region tend to be compressed and interconnected, forming defects with volumes larger than 10 mm^3^ [12]. These features, including pore size, morphology, orientation, and spatial aggregation, collectively determine the geometric anisotropy of pore defects in 3DPC and may significantly reduce the effective load-bearing area of the stressed cross-section [13].

To quantify the influence of pores on the strength of 3DPC, various empirical or semi-empirical prediction models have been developed based on porosity, pore size distribution, critical crack length, or macroscopic experimental data [14,15]. These models can, to some extent, describe the correlations between porosity and tensile or shear strength. However, they remain insufficient for revealing the independent effects of different pore geometric parameters on local stress concentration, crack initiation, and crack propagation paths. This limitation becomes particularly pronounced in multi-pore systems, where stress field interference, crack bridging, and crack path competition may occur among pores, making macroscopic failure no longer governed solely by porosity or the maximum pore size. Therefore, models based only on statistical regression or equivalent pore parameters are inadequate for providing a unified explanation of strength degradation mechanisms under complex pore distributions.

Numerical simulation provides an effective approach for analyzing pore-induced fracture. Among available methods, the phase-field method (PFM) has demonstrated distinctive theoretical advantages in simulating complex fracture problems in quasi-brittle materials in recent years [16,17]. By introducing a continuous phase-field variable that smoothly evolves from 0 to 1, this method represents discrete cracks and approximates complex fracture topologies through spatial volume integrals [18]. Unlike the traditional extended finite element method (XFEM), the PFM does not require explicit crack surface tracking and exhibits strong robustness in dealing with multi-pore interaction, multiple crack initiation, branching, and spatial coalescence [19]. Moreover, substantial progress has been achieved in the PFM regarding local adaptive mesh refinement and numerical solution stability [20]. Therefore, the phase-field cohesive zone model (PF-CZM) provides an appropriate numerical tool for investigating the mesoscopic fracture mechanisms induced by interlayer pores in 3DPC.

Although existing studies have recognized the detrimental effects of interfacial pores on the performance of 3DPC, a systematic understanding is still lacking regarding how the complex geometric characteristics and spatial arrangements of pores govern crack evolution and strength degradation. Accordingly, this study employs PF-CZM to establish controllable mesoscopic pore models and investigates the tensile failure behavior of 3DPC under single-pore, double-pore, and multi-pore conditions. The aim of this work is not to propose a new phase-field fracture theory, but rather to exploit the capability of PF-CZM in simulating crack initiation, propagation, coalescence, and path competition, thereby revealing the mechanisms by which interlayer pore geometries affect the tensile performance of 3DPC.

Therefore, compared with existing studies, the specific contributions of this work are summarized as follows:

1. Based on the typical morphological characteristics of interlayer pores in 3DPC, parameterized mesoscopic defect models are established, in which pore size, aspect ratio, inclination angle, spatial position, and relative multi-pore arrangement can be independently controlled.
2. PF-CZM is employed to capture the processes of crack initiation, propagation, bridging, coalescence, and path competition induced by multiple pores, thereby revealing the effects of effective pore projection length, connection angle, and local aggregation on tensile strength degradation.
3. A unified interpretive framework is proposed, extending from the “effective projected length of a single pore” to “combined defects and crack path competition in multi-pore systems”, providing a mesomechanical basis for strength prediction and printing process optimization of 3DPC.

## 2. Methodology

### 2.1. Phase-Field Method

#### 2.1.1. Theoretical Foundations

Fracture modeling based on the phase-field method (PFM) has matured into a robust and well-established theoretical paradigm for simulating crack initiation, propagation, and coalescence without the need for explicit crack tracking. In this study, an established phase-field cohesive zone model (PF-CZM), following the unified phase-field framework proposed by Wu et al. [16,21] and building upon our previous numerical implementations [20,22], is adopted to simulate pore-induced fracture in 3D-printed concrete. The objective is not to develop a new phase-field theory or computational framework, but to employ this robust fracture modeling approach to investigate how representative pore geometries and spatial arrangements affect stress concentration, crack evolution, and tensile strength degradation in 3DPC.

Inspired by mathematical concepts from image segmentation [23], the complex fracture topology can be approximated via a volumetric spatial integration [18]:

(1) $$\underset{\mathrm{Sharp}\;\mathrm{crack}}{\underbrace{A_{s}=\int_{S}dA}}=\underset{\mathrm{Regularized}\;\mathrm{crack}}{\underbrace{\int_{B}\gamma (d;\nabla d)=A_{d}(d)}}$$

In the equation, S signifies the discrete sharp crack surface, whereas B denotes the regularized smeared crack domain, where the original sharp discontinuity is diffused over a characteristic length scale B. To track the structural integrity, a continuous damage variable d (the phase field) is introduced. This parameter smoothly transitions between d=0 (representing entirely intact material) and d=1 (indicating a completely ruptured state). Following the formulation proposed by Ambrosio et al. [24], the crack surface density functional ∇d is expressed as follows:

(2) $$\left\{\begin{array}{l} \gamma (d;\nabla d)=\frac{1}{c_{a}}\left[\frac{1}{l}\alpha (d)+l{\left|\nabla d\right|}^{2}\right]\\ c_{a}=4\int_{0}^{1}\sqrt{\alpha (\beta )}d\beta \end{array}\right.$$

Furthermore, the geometric dissipation function α(d), which monotonically increases with respect to the damage variable within the interval [0, 1], is customarily constructed as follows:

(3) $$\alpha (d)=\xi d+(1-\xi )d^{2}$$

In the equation, ξ serves as a tunable scalar bounded between 0 and 2.

Governed by thermodynamic principles and the strict requirement of damage irreversibility, the evolution of the phase field within the physical domain Ω is dictated by the following [21]:

(4) $$\left\{\begin{array}{ll} \nabla \cdot q+Q\;(\varepsilon ,d)\leq 0, & \mathrm{in}\;\Omega \\ q\cdot n_{B}\geq 0, & \mathrm{on}\;\Omega \end{array}\right.$$

In the equation, the variables q and Q (ε,d) denote the spatial flux and the local source term of the phase field, respectively, whereas $n_{B}$ represents the outward unit normal vector of the boundary. Specifically, these components are expanded as follows:

(5) $$\left\{\begin{array}{l} q:=\frac{2l}{c_{a}}G_{f}\nabla d\\ Q\;(\varepsilon ,d)=-\omega^{\prime }(d)\bar{Y}-\frac{1}{c_{a}l}G_{f}\alpha^{\prime }(d) \end{array}\right.$$

In the equation, $G_{f}$ stands for the material’s critical fracture toughness, ε indicates the strain tensor, $\omega^{\prime }(d)$ signifies the derivative of the stiffness degradation function, and $\bar{Y}$ captures the active crack driving force. The energetic degradation ω(d) and driving forces $\bar{Y}$ are mathematically formulated as follows:

(6) $$\left\{\begin{array}{l} \bar{Y}:=\frac{\partial \psi }{\partial \omega }\\ \omega (d)=\frac{{\left(1-d\right)}^{r}}{{\left(1-d\right)}^{r}+a_{1}d\cdot (1+a_{2}\cdot d+a_{3}\cdot d^{2})} \end{array}\right.$$

In the equation, ψ indicates the elastic strain energy density of the pristine material, while r, $a_{1}$, $a_{2}$, and $a_{3}$ are empirical constants derived from experimental calibration.

Alongside the damage evolution, the mechanical displacement field (u) must satisfy the standard linear momentum balance:

(7) $$\left\{\begin{array}{ll} \nabla \cdot \sigma +b=0, & \mathrm{in}\;\Omega \\ \sigma \cdot n=t, & \mathrm{on}\;\partial \Omega \end{array}\right.$$

In the equation, σ is the Cauchy stress tensor, b is the applied body forces, and t is the prescribed surface tractions.

#### 2.1.2. Computational Strategy and Implementation

To facilitate finite element analysis, the strong forms of the governing equations (Equations (4) and (7)) are recast into their corresponding variational weak formulations:

(8) $$\left\{\begin{array}{l} \int_{\Omega }b\cdot \delta udV+\int_{\partial \Omega }t\cdot \delta udA=\int_{\Omega }\sigma :\nabla \delta udV\\ \int_{B}Q\delta ddV-\int_{B}q\cdot \nabla \delta dV\leq 0 \end{array}\right.$$

Applying standard Galerkin spatial discretization, the continuous fields (displacement and damage) alongside their gradients are interpolated using nodal values:

(9) $$\begin{array}{l} u(x)=Na,\;\varepsilon (x)=Ba\\ d(x)=\bar{N}\bar{a},\;\nabla d=\bar{B}\bar{a} \end{array}$$

In the equation, a and $\bar{a}$ denote the discrete nodal displacement and phase-field vectors, respectively. N and $\bar{N}$ represent the standard interpolation shape functions, whereas B and $\bar{B}$ map the spatial gradients (refer to [25] for explicit derivations).

The coupled nonlinear system is solved via an incremental step-by-step procedure. The total loading history is divided into M discrete pseudo-time increments (e.g., $\Delta t:=t_{m+1}-t_{m}$). During each step $t_{m+1}$, the unknown state variables are iteratively resolved based on the converged equilibrium states from the preceding step $t_{m}$.

To strictly prevent non-physical crack healing during unloading phases, a strain history functional H is introduced to replace the current elastic energy $\bar{Y}$:

(10) $$H=\max_{m\in [0,T]}(\bar{Y_{0}},{\bar{Y}}_{m+1})\to Q(\varepsilon ,d)=-\omega^{\prime }(d)H-\frac{G_{f}}{c_{a}l}\alpha^{\prime }(d)$$

In the equation, Y0¯=f2t2E0 establishes the critical energy barrier required to trigger initial damage.

By incorporating this history variable, the thermodynamically constrained inequality is naturally satisfied by the following:

(11) $$\left\{\begin{array}{l} r=f^{ext}-\int_{\Omega }B^{T}\sigma dV=0\\ \bar{r}=\int_{B}{\bar{N}}^{T}QdV-\int_{B}{\bar{B}}^{T}qdV=0 \end{array}\right.$$

To decouple the governing equations, a robust staggered algorithmic framework [17,26] is employed at each loading increment, executing the following sequence:

Mechanical step: Freezing the phase-field variable $\bar{a}={\bar{a}}^{\left(k-1\right)}$, the mechanical equilibrium is solved to obtain the updated displacements $a^{k}$ and corresponding stress $\sigma^{k}$:

(12) $$\left\{\begin{array}{l} \underbrace{K_{uu}\delta a^{k}=\int_{\Omega }B^{T}\omega (\bar{a})DBdV\delta a^{k}}=\underbrace{f^{ext}-\int_{\Omega }B^{T}\bar{\sigma }dV=r}\\ \bar{\sigma }=\omega (\bar{a})\cdot DBa^{k-1}\\ a^{k}=a^{k-1}+\delta a^{k}\\ \sigma_{k}=DBa^{k} \end{array}\right.$$

Damage step: Keeping the displacement field $a=a^{k}$ constant, the phase-field evolution equation is solved to update the damage state ${\bar{a}}^{k}$:

(13) $$\left\{\begin{array}{l} \underbrace{K_{dd}\delta {\bar{a}}^{k}=\int_{B}\left[{\bar{N}}^{T}\left(-\frac{\partial Q}{\partial d}\right)\bar{N}+\frac{2l}{c_{a}}G_{f}{\bar{B}}^{T}\bar{B}\right]dV\delta {\bar{a}}^{k}}=\underbrace{\int_{B}{\bar{N}}^{T}QdV-\int_{B}{\bar{B}}^{T}qdV=\bar{r}}\\ Q(\sigma_{k},{\bar{a}}^{\left(k-1\right)})=-\omega^{\prime }({\bar{a}}^{\left(k-1\right)})H^{k}-\frac{G_{f}}{c_{a}l}\alpha^{\prime }({\bar{a}}^{\left(k-1\right)})\\ H^{k}=\max\left(\frac{\max(f_{t},{\left[\sigma_{k}\right]}^{1})}{2E_{0}},H^{\left(k-1\right)}\right)\\ {\bar{a}}^{k}={\bar{a}}^{\left(k-1\right)}+\delta {\bar{a}}^{k} \end{array}\right.$$

Convergence verification: The iterative loop halts when the variation between successive damage increments ($\delta \bar{a^{k}}$) falls below a prescribed tolerance threshold; otherwise, the process advances to the next iteration.

The numerical simulations in this study were conducted utilizing a customized, in-house MATLAB 2024 phase-field solver [20,22,27], which has been specifically enhanced to accommodate corrosion-induced degradation phenomena. The source code is accessible from the corresponding author upon reasonable request.

### 2.2. Numerical Experiments

#### 2.2.1. Models and Parameters

To investigate the effects of pore characteristics on the macroscopic tensile performance and crack propagation mechanisms of 3D-printed concrete, a two-dimensional rectangular geometric model with dimensions of 15 mm × 20 mm was established in this section. The model dimensions and pore geometric parameters were selected based on the X-CT characterization results and mechanical test data summarized in the Introduction, aiming to provide a representative abstraction of the typical interlayer pore size, morphology, orientation, and local aggregation features in 3DPC, rather than an arbitrary configuration. The computational domain is sufficiently large to accommodate representative mesoscopic pores and their different arrangements, while maintaining a certain distance between the pores and the external boundaries to reduce boundary effects.

Based on the above considerations, this study does not further discuss the pore formation mechanism, but abstracts the real complex pores into parameterized two-dimensional elliptical defects from the perspective of numerical modeling. This simplified model preserves the major geometric features of interlayer pores in 3DPC, such as flattening, preferential orientation, and local aggregation, while facilitating a systematic investigation of the effects of pore size, aspect ratio, inclination angle, and spatial arrangement on crack initiation, propagation path, and tensile strength degradation. In addition, the elliptical pore model allows for independent control of key geometric variables, which helps identify the dominant pore parameters governing the tensile failure behavior of 3DPC.

It should be noted that pores in actual 3D-printed concrete usually exhibit irregular three-dimensional morphologies and may involve local connectivity or mutual coalescence. Therefore, the two-dimensional elliptical pore model adopted in this study is not intended to fully reconstruct the real pore network, but should be regarded as a representative idealized defect model. Nevertheless, this simplified approach provides a controllable analytical framework with clear physical meaning for revealing the intrinsic relationship between characteristic pore geometric parameters and the macroscopic tensile performance of 3DPC.

**Figure 1:**
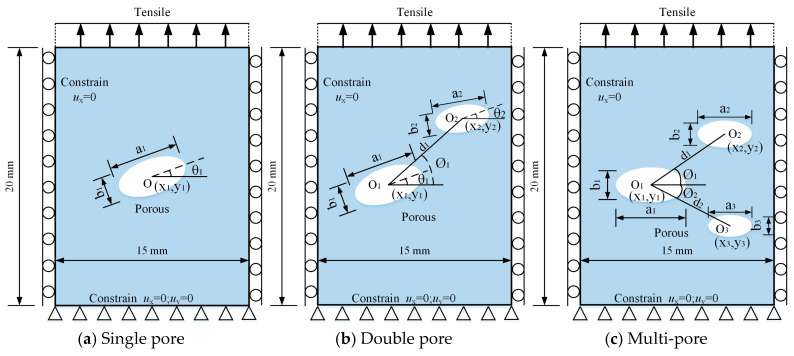
Geometric models of pores and loads.

Considering that the layer-by-layer forming process of 3D-printed concrete causes internal pores to be mainly concentrated at interlayer regions, such structural weakening leads to a significant reduction in tensile performance compared with conventional cast concrete and has become a key issue in both engineering applications and theoretical studies [28,29]. Therefore, a uniaxial tensile loading condition was adopted uniformly in this numerical study. The boundary conditions of the model were defined strictly according to the tensile stress state: the bottom boundary of the model was fully fixed $\left(u_{x}=0,u_{y}=0\right)$, the left and right boundaries were constrained in the horizontal normal direction $\left(u_{x}=0\right)$ to restrict lateral displacement, and a uniform vertical tensile load was applied to the top boundary of the model. The detailed pore geometric models and loading conditions used in the numerical tests are shown in Figure 1.

To ensure quasi-static equilibrium during the brittle fracture process, a displacement-controlled loading method was adopted, in which a uniform vertical tensile displacement was applied to the top boundary of the specimen instead of directly applying force-controlled loading. The bottom boundary was fixed in the vertical direction, and the top boundary was loaded incrementally along the vertical direction. The entire loading process was solved using an incremental step-by-step strategy, with the vertical displacement increment of each solution step set to $1\times 10^{-4}$ mm. This displacement increment is much smaller than the characteristic size of the specimen and the displacement variation scale during crack propagation, which can effectively avoid crack jumping and errors in capturing the peak load caused by an excessively large loading step, thereby ensuring that crack initiation, propagation, and coalescence are solved under approximately quasi-static conditions.

Given the complex pore morphology and irregular boundaries in actual 3D-printed concrete, the pores were idealized as elliptical defects in this study. This treatment not only preserves the essential flattened and elongated geometric characteristics of pores, but also effectively avoids mesh distortion and local stress singularity caused by microscopic rough boundaries, thereby improving the convergence and efficiency of numerical calculations. Meanwhile, elliptical pores can be described using a limited number of geometric parameters, which is beneficial for decoupling complex defect morphologies into several independent variables and conducting systematic parametric analyses.

In the single-pore model, pore size, aspect ratio, inclination angle, and spatial position were selected as the main variables, as shown in Figure 1a. Specifically, a single elliptical pore with the center coordinate $O_{1}(x_{1},y_{1})\;$was pre-embedded in the computational domain. The semi-major axis $a_{1}$ and semi-minor axis $b_{1}$ were used to characterize the absolute size of the pore; the aspect ratio $a_{1}/b_{1}$ was used to describe the morphological evolution of the pore from a nearly circular shape to a flattened elliptical shape; the major-axis inclination angle $\theta_{1}$ was used to characterize the orientation variation of the pore major axis relative to the loading direction or printing direction; and the pore center coordinate $O_{1}(x_{1},y_{1})$ was used to evaluate the positional sensitivity of an isolated defect in the tensile field. By varying these parameters, typical isolated pores formed due to extrusion effects and anisotropy can be systematically simulated.

Considering the realistic tendency of pores in 3D-printed concrete to aggregate and interconnect, double-pore and multi-pore models were further constructed to examine the synergistic effects among complex defects, as shown in Figure 1b. In the double-pore model, a second elliptical pore with the center located at $O_{2}(x_{2},y_{2})$ was introduced. The spatial arrangement between the two pores was described by the center-to-center distance $d_{1}$ and the positional angle $\Phi_{1}$ between the line connecting the two pore centers and the horizontal direction. Meanwhile, the relative size ratio $a_{2}/a_{1}$ was used to characterize the size gradation between the dominant large pore and the associated small pore, while the inclination angle combination $\left(\theta_{1},\theta_{2}\right)$ was used to analyze stress field interference and variations in crack propagation paths between pores with different orientations.

On this basis, the multi-pore model further introduced a third pore $O_{3}(x_{3},y_{3})$, together with multiple sets of relative distances $d_{1},d_{2}$ and positional angles $\Phi_{1},\Phi_{2}$. By flexibly combining the relative spatial positions, size differences, and major-axis inclination angles of different pores, the key mesoscopic defect characteristics affecting the tensile performance of 3DPC can be preserved while simplifying the geometric complexity of real pores. This makes it possible to further reveal the local stress field interference, crack bridging, and crack coalescence behavior in multi-pore systems under tensile loading.

#### 2.2.2. Numerical Experimental Scheme and Evaluation Criteria

**Figure 2:**
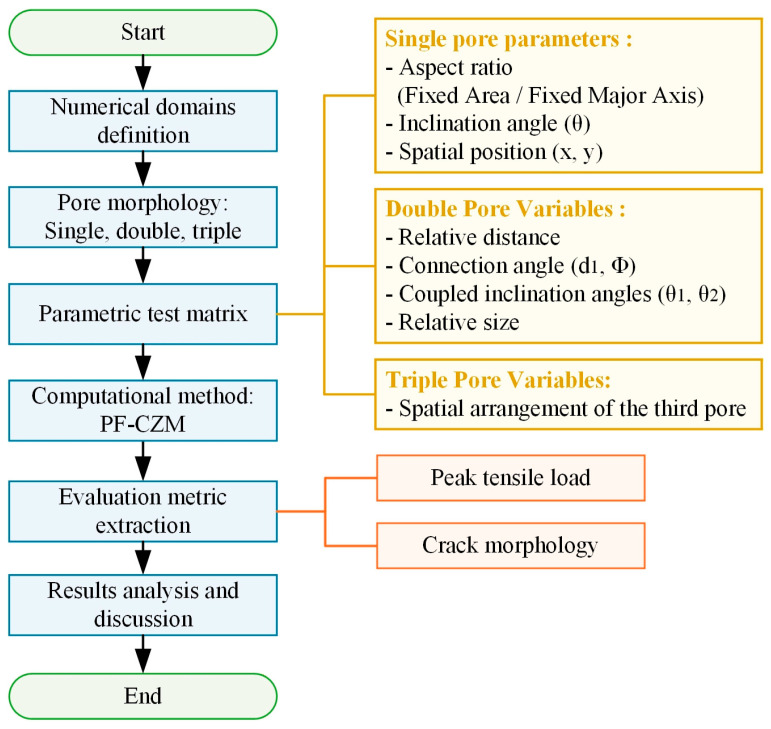
Research program and methodological flowchart.

To systematically carry out the numerical simulation research on the pore characteristics of 3DPC, this section formulates a comprehensive numerical experimental scheme and evaluation criteria based on the previously determined geometric models and pore parameters. As shown in Figure 2, the overall numerical calculation workflow first starts with establishing a unified two-dimensional rectangular computational domain, and then sequentially constructs the geometric morphologies of single-pore, double-pore, and multi-pore (three-pore) models according to the complexity of the defects.

After determining the pore category, the phase-field cohesive zone model (PF-CZM) is introduced as the core numerical method to simulate crack initiation, propagation, and coalescence from the pore tips under tensile loading. Compared with the conventional AT1 and AT2 phase-field models, PF-CZM can more reasonably describe crack nucleation and post-cracking strain-softening behavior in quasi-brittle materials. It has been widely used in numerical simulations of fracture processes in concrete and cement-based materials and has shown good capability in predicting crack paths in existing benchmark examples and experimental comparison studies.

For the model parameters, the softening coefficients were adopted from the values recommended by Wu Jianying et al. [30] in studies on PF-CZM, as listed in Table 1. It should be noted that the parameters in Table 1 are mainly used to describe the constitutive behavior, damage evolution, and softening response of concrete. The mechanical parameters of 3D-printed concrete, including elastic modulus, tensile strength, and fracture energy, were determined with reference to engineering measurements and existing literature, as summarized in Table 2 [29,31,32].

The purpose of adopting these parameters is to establish a representative baseline material model, rather than to perform inverse calibration for a specific batch or mix proportion of 3D-printed concrete specimens. Therefore, this study does not aim to predict the absolute tensile load-carrying capacity of a particular specimen. Instead, under identical material parameters, boundary conditions, and meshing strategies, only the pore geometric characteristics are varied, and the normalized peak tensile load, relative strength reduction, and crack propagation morphology are compared. This treatment helps reduce the influence of uncertainty in material parameters on the conclusions and highlights the relative effects of pore size, aspect ratio, inclination angle, spatial position, and multi-pore arrangement on the tensile failure mechanism.

To ensure the stability of the phase-field fracture simulation results, the relationship between mesh size and the regularization length scale was controlled. For PF-CZM, when the characteristic mesh size satisfies h≤l/5, the crack propagation path and load response generally exhibit good mesh independence. In this study, the regularization length was set as l=4.0 mm; therefore, the recommended maximum mesh size is l/5=0.8 mm. In the calculations, the initial mesh size was set to be smaller than 0.8 mm, and an adaptive mesh refinement method was employed in the MATLAB solver to locally refine the mesh near the pore tips and crack propagation regions [20,27]. Consequently, the effective mesh size in the critical damage regions satisfies the requirement of h≤l/5, ensuring the stability and reliability of the numerical results.

In the design of the numerical experiments, multiple sets of independent control variables are set for different pore categories. For the single-pore model, the influences of the aspect ratio (compared under two baselines: fixed pore area and fixed major axis length), major-axis inclination angle (θ), and spatial position (x, y) of the pore within the computational domain on the mechanical behavior are mainly investigated. For the double-pore model, the focus is on the interaction between pores, and the variables include the relative distance and connecting angle between the two pores (d_1_, Φ), the coupled inclination angle combinations of the double pores (θ_1_, θ_2_), and the size gradation effect. In the three-pore model, the focus further shifts to the interference and disturbance effect of the spatial arrangement of the third pore on the original double-pore stress field.

To scientifically quantify and compare the weakening degrees of different pore characteristics on the macroscopic and microscopic mechanical properties of 3DPC, this experiment uniformly extracts two key indicators as the final evaluation criteria. The first is the peak tensile load at the macroscopic level, which is used to intuitively evaluate the degradation amplitude of the material’s tensile bearing capacity under different defect distributions. The second is the crack morphology, which reveals the intrinsic mechanisms of crack evolution under different geometric parameters by extracting the final fracture modes. These two unified indicators will provide solid data support for the subsequent analysis of the experimental results and discussion of mechanisms.

## 3. Result

### 3.1. Effect of Single Pore Geometry on Strength

#### 3.1.1. Influence of Pore Morphology and Size Evolution

To analyze the influence of single-pore morphology on the tensile strength of 3D-printed concrete, two comparative scenarios were considered in this study. In the first scenario, the pore area was kept constant while only the aspect ratio $a_{1}/b_{1}$ was varied. In the second scenario, the horizontal semi-major axis $a_{1}$ was kept constant while the semi-minor axis $b_{1}$ was changed.

**Figure 3:**
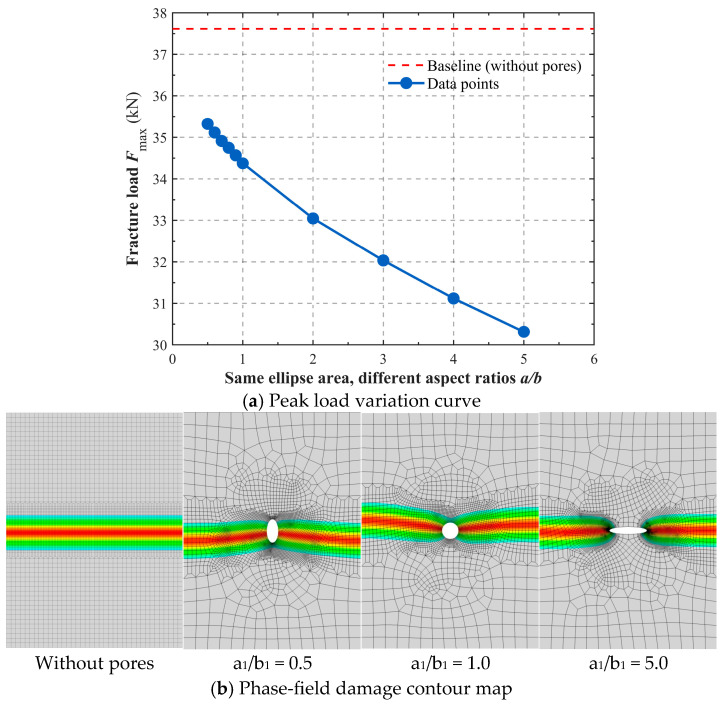
Effect of pore aspect ratio on structural performance under constant area. (**a**) Blue solid line with circles: peak load $F_{max}$ for a single elliptical pore; x-axis is $a_{1}/b_{1}$. Red dashed line: reference without pores. (**b**) Phase-field damage d: blue (low) to red (high); white indicates the elliptical pore.

Under the equal-area condition, the structural peak load decreased significantly with increasing aspect ratio $a_{1}/b_{1}$. As shown in Figure 3a, the peak load of the pore-free reference model was approximately 37.6 kN. When $a_{1}/b_{1}=0.5$, the peak load decreased to approximately 35.3 kN, corresponding to a reduction of about 6.1%. When the pore was circular, i.e., $a_{1}/b_{1}=1.0$, the peak load was approximately 34.4 kN, with a reduction of about 8.5%. As $a_{1}/b_{1}$ increased to 5.0, the peak load further decreased to approximately 30.3 kN, corresponding to a reduction of 19.4%. This indicates that, under the same pore area, a flatter pore with a more horizontal distribution causes a more pronounced weakening effect on the tensile load-bearing capacity.

The phase-field damage results in Figure 3b further explain the fracture mechanism underlying this trend. As $a_{1}/b_{1}$ increases, the pore gradually evolves from a nearly circular or vertically elliptical shape into a horizontally elongated defect. Its projected length perpendicular to the tensile direction increases, resulting in a reduction in the effective load-bearing cross-section. Meanwhile, stress concentration at both tips of the pore major axis becomes more significant, making cracks more likely to initiate from the pore tips and rapidly penetrate the specimen at a lower load level. Therefore, under the equal-area condition, the decrease in peak load is not governed solely by pore area, but is mainly related to the horizontal extension degree of the pore. Chen Wei et al. [33] also reported that the printing process makes pores more elongated, with the roundness value increasing from 0–5 in cast specimens to 15–90 in printed specimens. Elongated pores induce higher stress concentration under loading, whereas circular pores can disperse stress more uniformly. Yu Qian et al. [34] proposed that when the major axis of an ellipsoidal pore is parallel to the loading direction, the stress concentration around the pore is relatively low and the strength is the highest; when the minor axis of the ellipsoid is parallel to the loading direction, strong stress concentration occurs around the pore and the strength decreases significantly.

**Figure 4:**
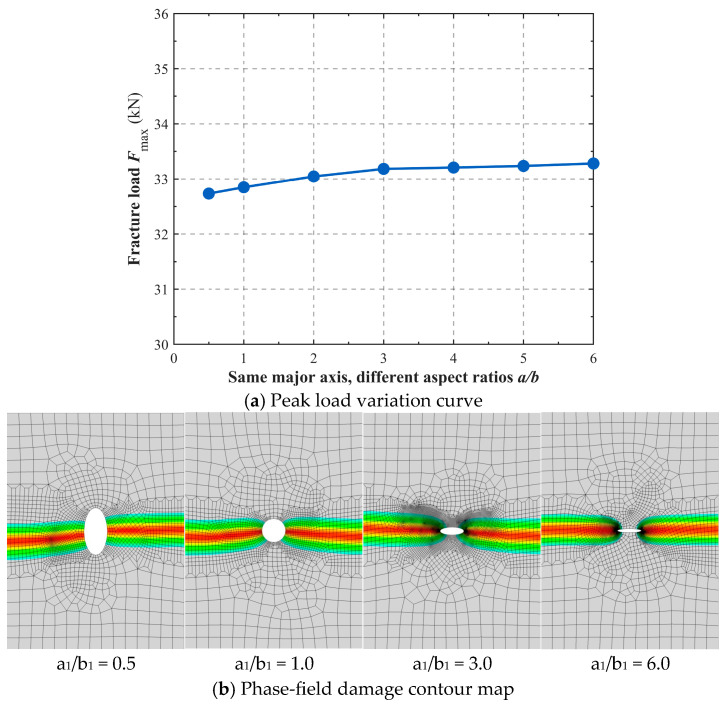
Effect of pore aspect ratio on structural performance under constant major axis. (**a**) Blue line with circles: peak load $F_{max}$ vs. pore aspect ratio a/b. (**b**) Phase-field damage d: blue (low) to red (high); white pore indicates the embedded pore; black lines are the computational mesh.

Furthermore, under the condition of a fixed horizontal semi-major axis $a_{1}$, the variation in peak load with the aspect ratio became much less pronounced. As shown in Figure 4a, the peak loads of all cases were lower than that of the pore-free reference model, but fluctuated only within a narrow range of approximately 32.7–33.3 kN, with a difference of about 0.6 kN. When $a_{1}/b_{1}=0.5$, the peak load was approximately 32.7 kN; when $a_{1}/b_{1}$ increased to 6.0, the peak load slightly increased to approximately 33.3 kN. Compared with the equal-area condition, this set of results indicates that when the horizontal projected length of the pore remains unchanged, merely changing the minor-axis length and local curvature does not cause a significant variation in load-bearing capacity.

Combined with the phase-field damage contours in Figure 4b, it can be seen that, in this group of cases, the projected length of the pore perpendicular to the tensile direction remains unchanged. Therefore, the crack initiation position and the penetration span are basically restricted within a similar range, and the effective load-bearing width of the structural cross-section is weakened to approximately the same extent. As $a_{1}/b_{1}$ increases, the pore gradually evolves from a vertical ellipse or nearly circular shape into a horizontally elongated morphology. However, the semi-minor axis $b_{1}$ continuously decreases, and the missing material area is correspondingly reduced, allowing part of the local stiffness to be retained. Consequently, no obvious decrease in peak load is observed.

Compared with the equal-area condition, this result suggests that the strength degradation caused by a single pore is not controlled by the aspect ratio alone. For a uniaxial tensile problem, the more critical geometric quantity is the transverse projected length of the pore on the loaded cross-section, namely, the extent to which it weakens the effective load-bearing section. The effects of pore area and local curvature play only secondary modulation roles.

#### 3.1.2. Influence of Pore Inclination Angle

**Figure 5:**
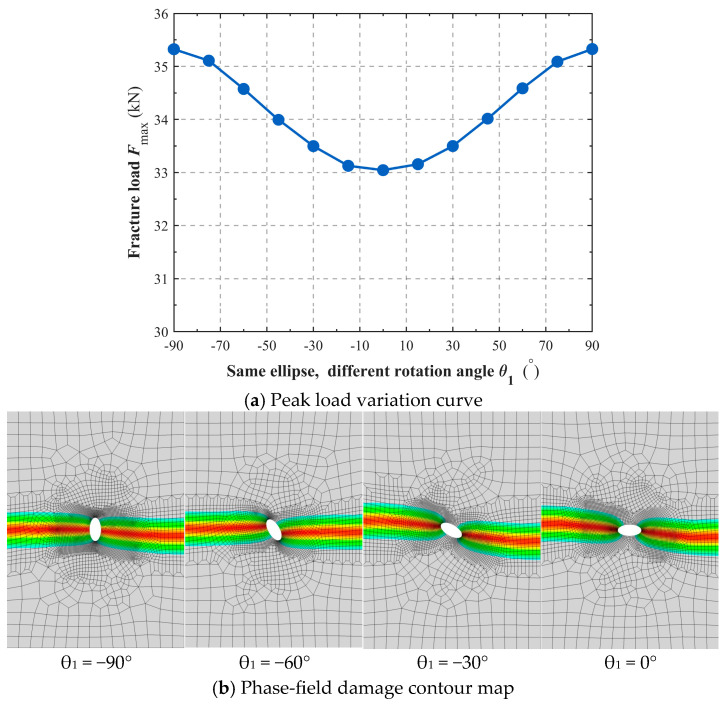
Effect of pore inclination angle on structural performance.

With the pore size and shape kept unchanged, the influence of the pore major-axis inclination angle $\theta_{1}$ on the structural peak load and crack path was further investigated. The calculation results are shown in Figure 5a. The peak load exhibits a clear symmetry with respect to $\theta_{1}$. When $\theta_{1}=\pm 90^{\circ }$, the pore major axis is parallel to the tensile direction, and the peak load reaches its maximum value of approximately 35.3 kN, corresponding to a reduction of about 6.1% compared with the pore-free reference model. As the pore rotates toward the horizontal direction, the peak load gradually decreases. When $\theta_{1}=0^{\circ }$, the pore major axis is perpendicular to the tensile direction, and the peak load reaches its minimum value of approximately 33.1 kN, corresponding to a reduction of about 12.0%.

This trend can still be explained by the effective projected length. As shown in Figure 5b, when $\theta_{1}=0^{\circ }$, the pore has the largest projection on the transverse load-bearing section, leading to the most severe reduction in effective load-bearing area. Meanwhile, both ends of the horizontal major axis become the main stress concentration regions, where cracks preferentially initiate and propagate toward both sides. As $|\theta_{1}|$ increases, the transverse projected length of the pore gradually decreases, the continuity of the cross-section is restored, and the stress concentration at the pore tips is correspondingly weakened. As a result, the peak load increases.

This result indicates that pore orientation is an important factor affecting the tensile anisotropy of 3DPC. If extrusion and interlayer deposition during printing cause flattened pores to be preferentially arranged horizontally, their weakening effect on vertical tensile load-bearing capacity becomes the most unfavorable. In contrast, when the pore major axis deviates from the horizontal plane, its weakening effect is significantly reduced. Jia Ru et al. [35] also revealed that the oriented distribution of pores inside 3D-printed concrete, especially the orientation of interlayer pores, is a key microscopic cause of mechanical anisotropy. When the pore direction is perpendicular to the loading direction, the effective load-bearing area is larger and the strength is higher; whereas when the pore direction is parallel to the loading direction, stress concentration occurs earlier, leading to premature failure. Therefore, in practical 3D printing engineering, if the extrusion process causes flattened pores to be preferentially distributed horizontally *θ*_1_ ≈ 0°, the overall tensile performance of the structure will be weakened most severely compared with other inclination angles.

#### 3.1.3. Influence of Pore Spatial Position Distribution

**Figure 6:**
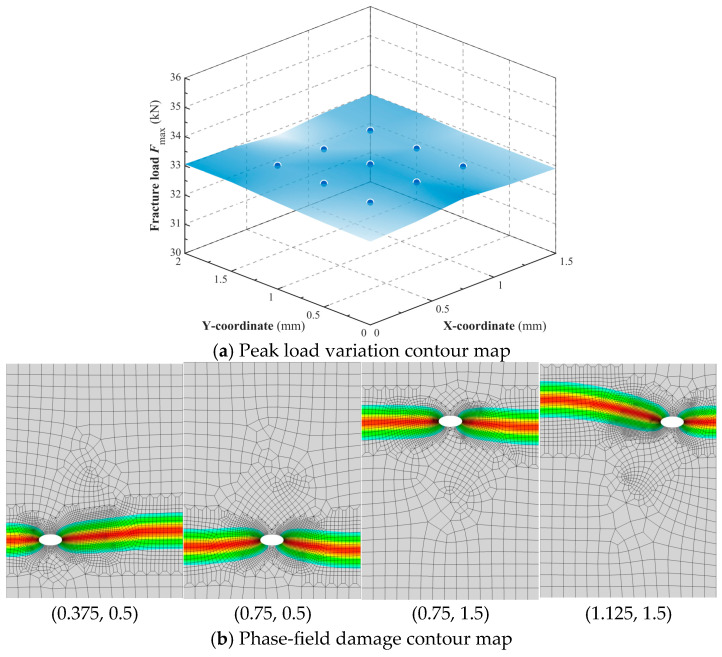
Effect of pore spatial position distribution on structural performance.

With the pore size, shape, and inclination angle fixed, the effects of variations in the pore center position $\left(x_{1},y_{1}\right)$ on peak load and crack path were examined. As shown in Figure 6a, when the pore translates inside the model, the structural peak load remains basically stable at approximately 33.1 kN, which is about 12.0% lower than that of the pore-free reference model. The differences among different position cases are very small, indicating that the spatial position sensitivity of a single pore in a uniform uniaxial tensile field is relatively weak.

The damage morphologies in Figure 6b show that changing the pore position mainly shifts the final crack penetration location, but does not alter the crack initiation mechanism. Since the pore size, inclination angle, and transverse projected length remain unchanged in all cases, the weakening effect of the pore on the effective load-bearing cross-section is the same, and the local stress concentration level at the pore tips is also basically identical. Therefore, provided that the pore is far from the external boundaries and the interlayer material heterogeneity is not considered, simple translation of a single pore does not significantly change the macroscopic peak load.

#### 3.1.4. Unified Interpretation of Single-Pore-Induced Strength Degradation

**Figure 7:**
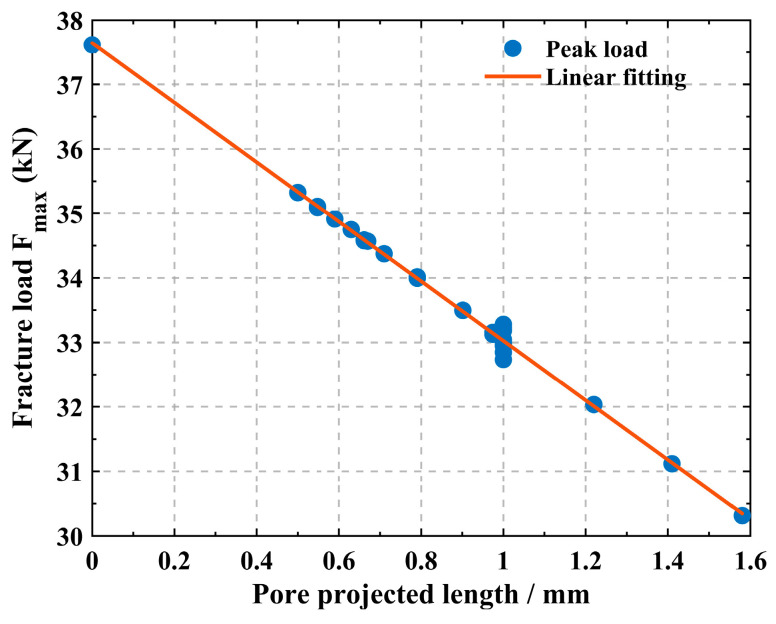
Relationship between peak tensile load and effective projected pore length perpendicular to tensile loading direction: Blue circles with a line: peak tensile load $F_{max}$ vs. effective projected pore length. Orange line: linear fitting of the data. x-axis: pore projected length (mm); y-axis: $F_{max}$ (kN).

The above results demonstrate that although the effects of pore aspect ratio, size, and inclination angle on peak load appear in different forms, their essence is closely related to the effective projected length of the pore perpendicular to the tensile direction. To provide a unified description of the influence of single-pore geometric characteristics on strength degradation, the relationship between the peak tensile load $F_{max}$ and the effective projected length $L_{p}$ was further established, as shown in Figure 7.

For an elliptical pore with a major-axis inclination angle $\theta_{1}$, semi-major axis $a_{1}$, and semi-minor axis $b_{1}$, its effective projected length perpendicular to the tensile direction can be approximately expressed as follows:

(14) $$L_{p}=2\sqrt{a_{1}^{2}\cos^{2}\theta_{1}+b_{1}^{2}\sin^{2}\theta_{1}}$$

where $\theta_{1}=0^{\circ }$ denotes a horizontal pore major axis, while $\theta_{1}=90^{\circ }$ denotes a vertical pore major axis. This expression reflects the extent to which the pore obstructs the load transfer path on the tensile cross-section.

The results in Figure 7 show that although the pore area, aspect ratio, and inclination angle differ among the various cases, the peak load data can still be reasonably collapsed onto a decreasing trend with increasing $L_{p}$. This indicates that $L_{p}$ can serve as the dominant geometric parameter characterizing the weakening effect of a single interlayer pore on the tensile strength of 3DPC. As $L_{p}$ increases, the intact ligament region available for stress transfer in the specimen decreases, and stress concentration near the pore tips becomes more pronounced. Consequently, cracks are more likely to initiate and penetrate at lower external loads. Therefore, the strength degradation mechanism under the single-pore condition can be summarized as follows: the larger the transverse projection of the pore, the stronger the weakening effect on the tensile cross-section, the lower the load required for crack initiation, and the smaller the final peak load-bearing capacity.

### 3.2. Effect of Double-Pore Interaction on Strength

#### 3.2.1. Effect of Relative Position and Connection Angle of Pores

Based on the single-pore analysis, a double-pore model was further introduced to investigate the effects of pore spacing $d_{1}$ and the connection angle Φ_1_, defined as the angle between the line connecting the centers of the two pores and the horizontal direction, on the tensile strength and crack propagation path.

**Figure 8:**
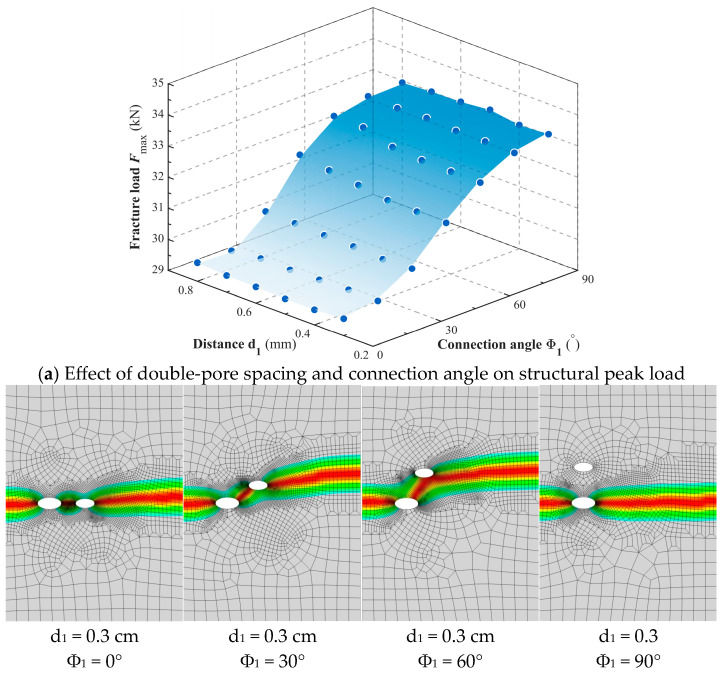

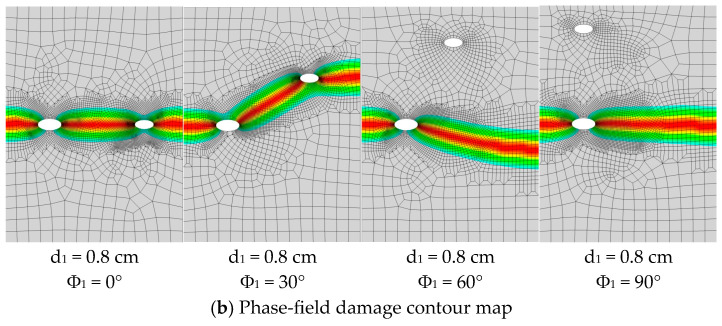
Effect of relative position and connection angle on specimen peak load and crack morphology under double-pore conditions. (**a**) Top: 3D surface of peak load $F_{max}$ (kN) vs. pore distance $d_{1}\;$(mm) and connection angle $\Phi_{1}\;$(°); blue dots are simulation points and the blue surface is the interpolated trend. (**b**) Crack-growth contours for different $d_{1}$ and $\Phi_{1}$: white ovals are the two pores; the color field shows phase-field damage d (blue/green: low to intermediate damage, red: high damage); black lines are the mesh.

As shown in Figure 8a, the presence of double pores further reduces the peak load of the specimen. However, the degree of strength reduction is mainly governed by the connection angle Φ_1_, whereas the influence of pore spacing $d_{1}$ is relatively limited. When $\Phi_{1}=0^{\circ }$, the two pores are horizontally collinear, and the peak load decreases to the minimum value of approximately 29.2 kN, which is significantly lower than that of the single-pore case. As Φ_1_ increases, the peak load gradually recovers. When Φ_1_ approaches $90^{\circ }$, the two pores are arranged nearly vertically, and the peak load stabilizes at approximately 34.5 kN. In contrast, varying $d_{1}$ under the same Φ_1_ does not lead to a pronounced difference in peak load, indicating that, within the parameter range considered in this study, the connection angle plays a more dominant role than pore spacing in controlling the strength degradation induced by double pores.

The phase-field damage contours in Figure 8b reveal the fracture mechanism underlying this trend. When Φ_1_ is small, especially at $\Phi_{1}=0^{\circ }$, the projections of the two pores perpendicular to the tensile direction overlap with each other, and the solid ligament between the pores becomes a region of high stress concentration. Cracks preferentially initiate between the two pores and rapidly bridge them, forming a transversely continuous defect and thereby causing a significant decrease in peak load. When Φ_1_ increases to 60–$90^{\circ }$, the transverse projections of the two pores become gradually staggered, and the coupling of their stress fields is weakened. In this case, the main crack is usually controlled by a single most unfavorable pore, while the other pore is less likely to participate in the through-fracture process due to local stress release. Therefore, the strength degradation of the double-pore system essentially depends on whether the two pores can form a continuous weakened band along the loaded cross-section.

This result indicates that, during the 3D printing process, it is necessary not only to reduce the overall porosity, but also to avoid the horizontal collinear aggregation of interlayer air voids at the same height. Such an arrangement significantly increases the equivalent transverse projected length of defects and promotes low-energy brittle through-fracture.

#### 3.2.2. Effect of Self-Inclination Angle and Connection Angle

**Figure 9:**
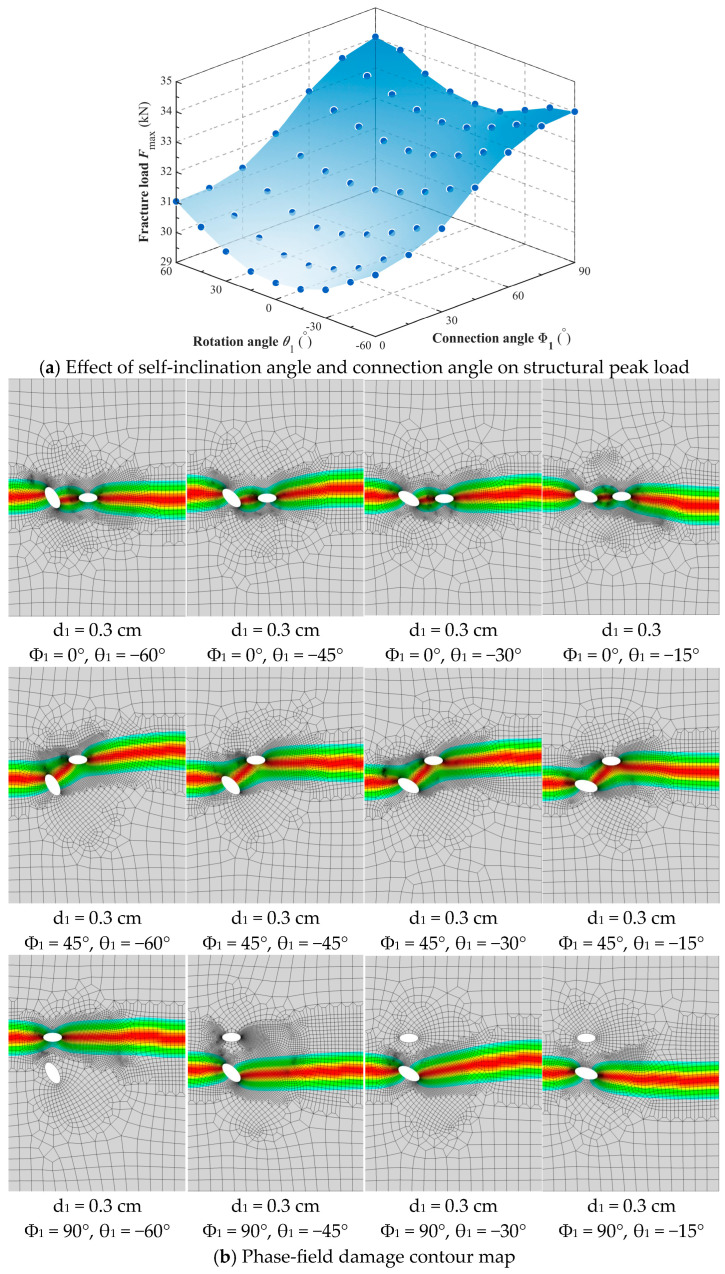
Peak load and crack morphology of double-pore specimens under different self-inclination angles and connection angles. (**a**) Top: 3D surface of peak load $F_{max}$ (kN) vs. rotation angle θ1 (°) and connection angle $\Phi_{1}\;$(°); blue dots are simulation points and the blue surface is the interpolated trend. (**b**) Crack-growth contours for different θ1 and $\Phi_{1}$: white ovals are the two pores; the color field shows phase-field damage d (blue/green: low to intermediate damage, red: high damage); black lines are the mesh.

With the pore spacing fixed at d_1_ = 0.3 cm and the right pore kept horizontal, the coupled effects of the inclination angle θ_1_ of the left pore and the connection angle Φ_1_ were further investigated. The calculation results are shown in Figure 9a. The peak load is affected by both angular parameters, but the connection angle Φ_1_ remains the dominant factor.

When Φ_1_ = 0° and the left pore is nearly horizontal, the transverse projections of the two pores and the stress concentration zones at their tips overlap with each other, reducing the peak load to approximately 29.0 kN. As Φ_1_ increases, the tendency for transverse connection between the pores becomes weaker, and the peak load generally recovers. For a fixed Φ_1_, the peak load increases with increasing |θ_1_|, which is consistent with the single-pore inclination analysis. That is, when the major axis of the pore deviates from the horizontal direction, its weakening effect on the tensile cross-section is reduced.

The phase-field damage contours in Figure 9b further show that crack bridging between the two pores is more likely to occur when the connection angle is small. In this situation, the inclination angle of the left pore mainly modifies the deflection path of the crack as it propagates from the left pore toward the right pore. When the left pore remains horizontal or nearly horizontal, the crack propagates along the line connecting the two pores, resulting in a short fracture path and low energy consumption. When the left pore is significantly inclined, the crack must deflect or kink before bridging the two pores, which increases the fracture path length and energy dissipation, thereby increasing the peak load. When Φ_1_ increases to 90°, the transverse projections of the two pores are no longer continuous, and the main crack is mostly controlled by a single pore. As a result, the synergistic failure effect of the double pores is markedly weakened. Therefore, the influence of pore inclination is mainly reflected in the modulation of the local crack path, whereas the connection angle determines whether the two pores can form a macroscopically continuous defect.

#### 3.2.3. Effect of Inclination Angle Combinations of Double Pores

Under the condition that the two pores are horizontally collinear, namely Φ_1_ = 0° and d_1_ = 0.3 cm, the influence of the inclination angle combination θ_1_, θ_2_ of the left and right pores on tensile strength was further analyzed.

**Figure 10:**
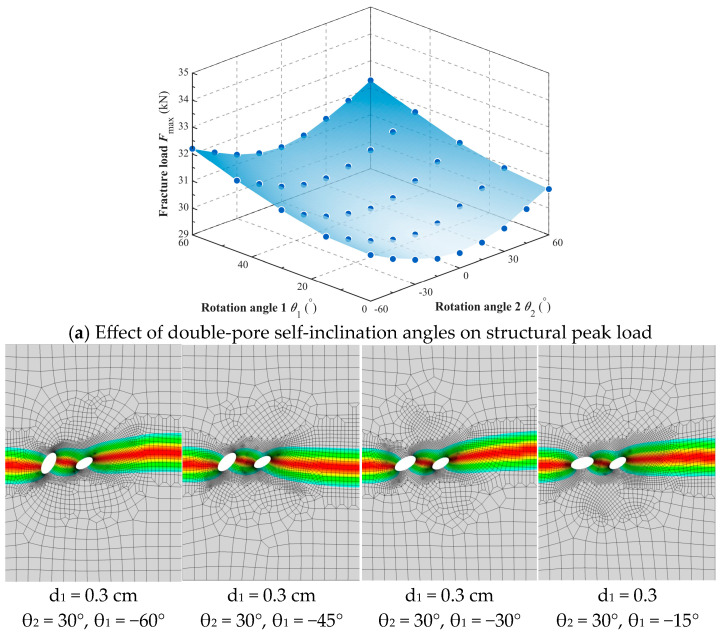

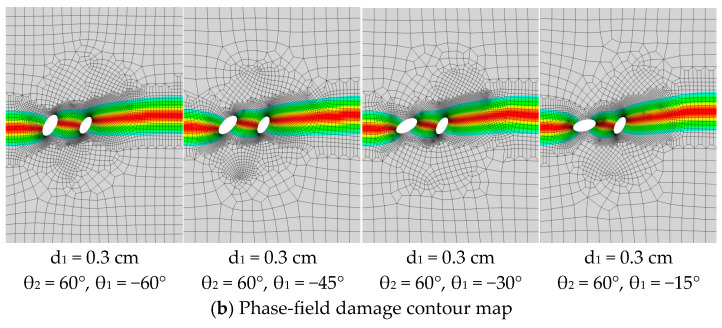
Peak load and crack morphology of double-pore specimens under different combinations of pore inclination angles. (**a**) Top: 3D surface of peak load $F_{max}$ (kN) vs. rotation angle θ1 (°) and rotation angle θ2 (°); blue dots are simulation points and the blue surface is the interpolated trend. (**b**) Crack-growth contours for different θ1 and θ2: white ovals are the two pores; the color field shows phase-field damage d (blue/green: low to intermediate damage, red: high damage); black lines are the mesh.

As shown in Figure 10a, when both pores are nearly horizontally oriented, the peak load reaches its lowest level, below 30.0 kN. This is because the two horizontally flattened pores have the largest transverse projections on the loaded cross-section, and the ligament region between the two pores is subjected to strong stress concentration, making rapid crack penetration highly likely. As |θ_1_| and |θ_2_| increase, the pores gradually deviate from the horizontal orientation, and the peak load shows a steady increasing trend. When the inclination angles of both pores increase to approximately 60°, the peak load can recover to above 32.5 kN.

The damage morphologies in Figure 10b show that, under the horizontally collinear arrangement, the main crack still tends to connect the two pores overall, but the pore inclination angles change the crack initiation points and propagation directions. When the pore inclination angles are small, the crack penetrates along an approximately straight path, and the structure exhibits typical brittle failure. When the pores are inclined, the maximum stress concentration points deviate from the horizontal axis, and the crack needs to deflect or kink before bridging the two pores, thereby increasing the fracture path and energy dissipation. Therefore, randomization of pore inclination or a non-coplanar pore arrangement can, to some extent, mitigate the adverse effect caused by horizontally collinear pores.

#### 3.2.4. Effect of Size Combination of Large and Small Pore

Under the condition that the two pores are horizontally collinear, with Φ_1_ = 0°, and the size of the primary pore is kept constant at a_1_ = 1.0 mm, the effect of the secondary pore size a_2_ on structural strength was investigated.

**Figure 11:**
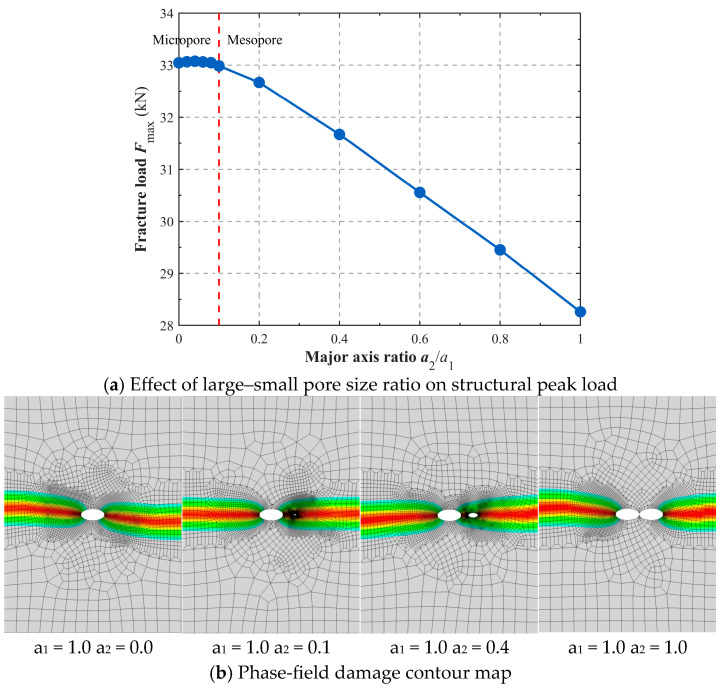
Peak load and crack morphology of double-pore specimens with different pore size ratios. (**a**) Blue line with filled circles: peak load $F_{max}$ vs. pore-size ratio $a_{2}/a_{1}$. Red dashed vertical lines indicate the micro-pore boundary. (**b**) Phase-field damage contour maps: white circles are pores; the color field shows damage variable d (blue: low, green/yellow: intermediate, red: high/cracked); black lines are the mesh.

As shown in Figure 11a, the peak load exhibits a nonlinear trend of “initial stability followed by decline” as the size ratio a_2_/a_1_ increases. When a_2_ ≤ 0.1 mm, the peak load remains nearly constant at approximately 33.1 kN, indicating that a very small secondary pore has a limited influence on the overall load-bearing capacity. When a_2_ > 0.1 mm, the peak load decreases markedly with increasing a_2_/a_1_. When the two pores become comparable in size, the peak load decreases to approximately 28.2 kN, suggesting that the secondary pore has transformed from a local disturbance source into an effective defect capable of synergistic failure with the primary pore.

The phase-field damage contours in Figure 11b explain this size threshold effect. For very small pores, the stress disturbance range is limited, making it difficult for them to effectively couple with the primary pore. Failure is still mainly governed by crack initiation at the tip of the primary pore. As a_2_ increases, both the transverse projected length and the stress concentration zone of the two pores expand, making crack bridging between the pores easier and eventually forming a through-crack. This result indicates that whether a secondary pore affects macroscopic strength depends not only on its existence, but more importantly on whether its size is sufficient to enter the stress-interference range of the primary pore.

Combined with the phase-field damage contours in Figure 11b, the nonlinear evolution mechanism can be further clarified. In the mesopore regime, namely a_2_ > 0.1 mm, the continuous extension of the major axis of the secondary pore sharply increases the total projected area of the two pores perpendicular to the tensile direction, resulting in severe weakening of the effective horizontal load-bearing cross-section. Meanwhile, the stress fields at the pore tips interfere with each other, allowing the main crack to readily penetrate between the two pores. In contrast, in the micropore regime, namely a_2_ ≤ 0.1 mm, the secondary pore degenerates into an almost point-like microdefect, and its disturbance to the surrounding stress field is extremely limited. At this stage, the micropore cannot form effective stress field coupling with the primary pore. Structural failure is completely dominated by the tip stress concentration of the primary pore, while the secondary micropore is only passively crossed by the main crack and therefore does not cause a substantial additional reduction in macroscopic load-bearing capacity.

Previous studies have also indicated that even when small pores are numerous, their influence on strength is much lower than that of aggregated and connected large pores if they are sufficiently small and isolated [36]. This result not only demonstrates that strictly controlling pore size and reducing the effective projected length of pores are crucial for maintaining structural strength during 3D printing, but also provides a basis for simplifying subsequent detection and simulation work. Since very small pores, for example those smaller than 0.1 mm, have negligible influence on macroscopic load-bearing capacity, extremely high resolution may not be necessary in CT scanning of internal pores. Similarly, such microstructures may be ignored when constructing numerical models, thereby effectively reducing computational cost and modeling complexity.

### 3.3. Effect of Multi-Pore Distribution on Strength

Under the complex baseline condition in which the first two pores were fixed in a non-collinear inclined arrangement, with the connection angle Φ_1_ = 60°, the influence of the position angle of the third pore, Φ_2_, varying from −90° to 90°, on the mechanical response and crack evolution path of the structure was further investigated.

**Figure 12:**
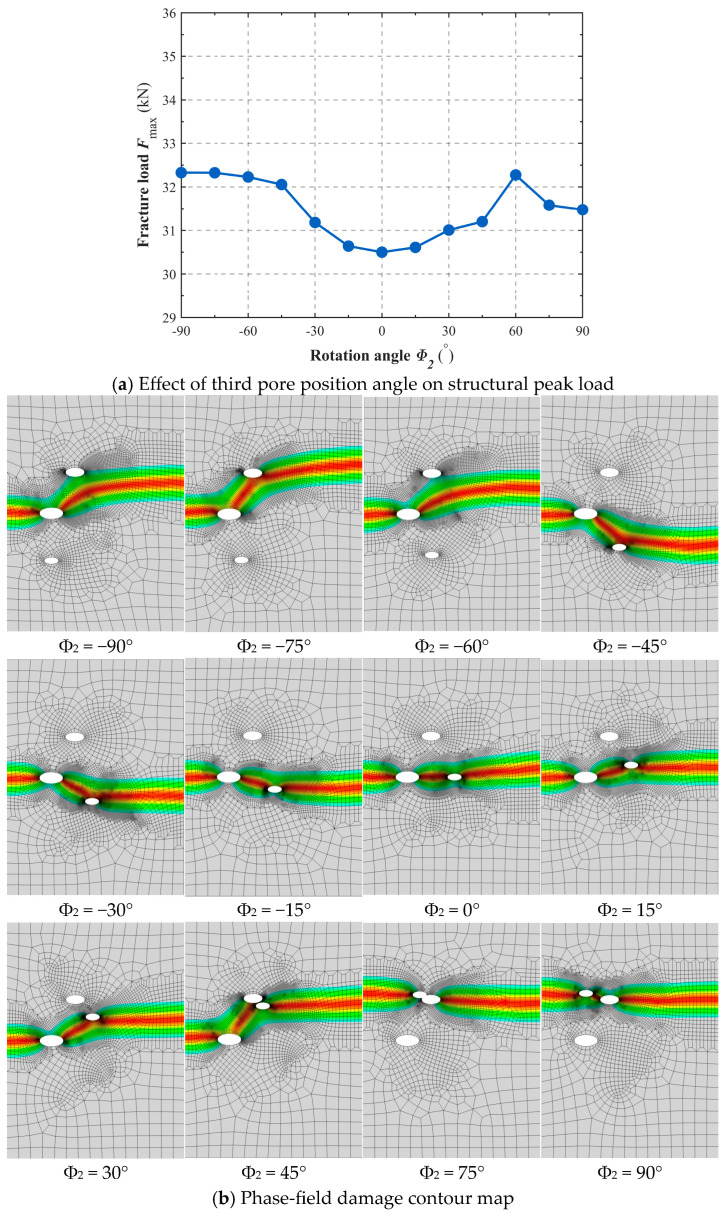
Peak load and crack morphology of multi-pore specimens under different third pore positions. (**a**) Blue line with filled circles: structural peak load $F_{max}$ (kN) versus the rotation angle $\Phi_{2}$ (°). x-axis: $\Phi_{2}$ from $-90^{\circ }$ to $90^{\circ }$. (**b**) Phase-field damage contour maps: white circles are the pores; the damage variable d uses blue (low/undamaged) to green/yellow (intermediate) to red (high/cracked); black lines indicate the computational mesh.

As shown by the numerical results in Figure 12a, the peak load of the structure exhibits a pronounced concave trend with the variation of Φ_2_. The quantitative data indicate that when the position angle of the third pore is close to Φ_2_ = 0°, the load-bearing capacity of the structure decreases to its minimum. This is mainly because, in this configuration, the third pore and the first primary pore form a highly unfavorable horizontally collinear arrangement. Such a local horizontal defect combination, which is perpendicular to the tensile loading direction, directly governs structural failure and severely weakens the effective load-bearing section in the middle region. As Φ_2_ gradually increases toward both sides, namely as the third pore deviates from the horizontal axis toward the vertical direction, the overall peak load shows a clear recovery trend. When the position angle increases to the range of ±60° to ±90°, the peak load recovers to a relatively high level.

Combined with the phase-field damage contours in Figure 12b, the crack path competition mechanism in the multi-defect system can be clearly revealed. When Φ_2_ is close to 0°, such as −15° and 0°, the horizontal stress concentration between pore 1 and pore 3 becomes absolutely dominant, and the main crack connects these two pores along an almost straight path, while bypassing pore 2 located in the upper-right region. When Φ_2_ lies in the lower half-plane, for example from −90° to −45°, the third pore deviates from the core stressed region, and its ability to guide the main crack is greatly weakened. In this case, the fracture path is again governed by the 60° inclined connection between pore 1 and pore 2.

It is worth noting that when Φ_2_ is within the range of 75° to 90°, the second and third pores become extremely close in space, and their stress fields strongly interact to form a new combined defect unit. The effective projected length of this unit exceeds that of the left primary pore, making it the weakest load-bearing region. According to the principle of minimum energy dissipation, the main crack preferentially initiates and propagates between the two smaller pores, completely bypassing the primary pore. Since this local fracture path requires lower energy consumption, the structural peak load decreases noticeably in this range, which also explains the asymmetry of the macroscopic load curve from a mechanistic perspective.

In summary, the macroscopic failure of a complex multi-defect system is not simply controlled by the single largest pore, but is governed by crack path competition induced by the spatial arrangement of multiple pores. Zhang et al. [37] also reported, based on X-CT analysis, that the pressure exerted by upper printed layers causes pores at the lower interface to become interconnected or continuously distributed, resulting in the lowest interfacial bonding strength. Therefore, local horizontal collinearity or dense aggregation of pores can significantly amplify the effective projected length of defects and consequently become a decisive factor inducing low-energy fracture of the structure.

## 4. Discussion

Although the phase-field fracture model adopted in this study is not a newly proposed numerical theory, the novelty of this work lies primarily in its mesoscopic application to pore-induced fracture in 3D-printed concrete and in the mechanistic interpretation derived from it. Existing studies on pore defects in 3DPC have mostly relied on X-CT-based statistical parameters, empirical relationships between porosity and strength, or equivalent defect models. These studies have effectively revealed the adverse influence of porosity on macroscopic mechanical performance; however, they generally have difficulty explicitly clarifying how the geometric parameters of individual pores and the interactions among multiple pores control crack initiation, bridging, coalescence, and final fracture path selection.

In contrast, this study establishes a controllable parametric analysis framework based on PF-CZM, in which pore size, aspect ratio, inclination angle, spatial position, connection angle, spacing, and size gradation can be varied independently. Therefore, the strength degradation of 3DPC can be interpreted not only in terms of overall porosity or defect area, but also from mesoscopic geometric mechanisms such as effective projected length, collinear pore arrangement, and the formation of combined defects. The main contribution of this study is the identification of the dominant geometric descriptors governing tensile failure: the effective projected length under the single-pore condition, the connection angle under the double-pore condition, and the crack path competition mechanism induced by local pore aggregation under the multi-pore condition.

It should be noted that, to highlight the influence of pore geometric parameters on the tensile performance and crack evolution behavior of 3DPC, the real complex pores were simplified as regular two-dimensional elliptical defects in this study. This treatment facilitates controllable single-factor parametric analysis, allowing key variables such as pore size, aspect ratio, inclination angle, spatial position, and arrangement pattern to be independently regulated. As a result, the dominant geometric factors affecting tensile strength degradation and crack propagation paths in 3DPC can be identified more clearly.

However, this simplified model cannot fully represent the complex features of pores in actual 3D-printed concrete, such as irregular pore boundaries, three-dimensional spatial morphology, local connectivity, and random spatial distribution. In particular, during the real printing process, the combined effects of material extrusion, interlayer overlap, bubble retention, and insufficient interfacial bonding may lead to irregular pore morphologies and local interconnection with neighboring pores, thereby generating more complicated stress concentration zones and crack propagation paths. Therefore, the numerical results presented in this study are mainly intended to reveal the fundamental laws and intrinsic mechanisms by which different pore parameters affect tensile behavior, rather than to fully reproduce the pore network and failure process of a specific physical specimen.

Future studies will combine X-ray computed tomography (X-CT) or image reconstruction techniques to obtain the real pore structures of 3DPC, establish numerical models corresponding to the actual pore morphology and spatial distribution of physical specimens, and further conduct quantitative validation by comparing numerical results with experimental observations. By introducing real three-dimensional pore networks, the effects of pore irregularity, connectivity, and random distribution on the tensile performance and fracture evolution behavior of 3DPC can be further evaluated, thereby improving the capability of the model to characterize the damage and failure processes of actual printed components.

With regard to the assumptions in fracture simulation, PF-CZM was employed in this study to describe the tensile fracture process of 3DPC under quasi-static tensile conditions. This model can simulate crack initiation, propagation, deflection, and coalescence of multiple cracks without predefining crack paths, making it suitable for analyzing pore-induced stress concentration and crack path competition. Considering that the focus of this study is the influence of interlayer pore geometric characteristics on tensile failure, aggregate gradation, fiber bridging, material time dependence, and complete anisotropic constitutive differences caused by printing paths were not explicitly considered. Instead, the material was treated as an equivalent quasi-brittle medium, and the main source of mesoscopic heterogeneity was represented by explicitly introduced pore defects.

This assumption is helpful for identifying the independent effects of pore geometric parameters under controlled-variable conditions. Nevertheless, it also means that the conclusions of this study are mainly applicable to explaining the relative strength degradation mechanism dominated by pore defects, rather than being directly interpreted as a complete prediction of the absolute load-bearing capacity of all actual 3DPC components.

## 5. Conclusions

Based on the phase-field method, this study systematically investigates the weakening mechanism and local crack evolution laws of interlayer pore characteristics in 3D-printed concrete (3DPC), including size, morphology, spatial orientation, and multi-pore synergistic arrangement, on its macroscopic tensile performance. By establishing a two-dimensional mesoscopic fracture model and conducting single-factor variable analysis, the following main conclusions are drawn:

(1) The degree of weakening of the macroscopic tensile strength of 3DPC caused by a single pore is controlled by the effective projected length of the pore in the direction perpendicular to the principal tensile stress, while it is insensitive to the spatial translational position of the pore. Flat pores with large aspect ratios and horizontal orientation lead to the most severe stress concentration and cross-sectional area reduction. Conversely, if the manufacturing process can induce a partial deflection of large pores, the performance degradation caused by single pores can be significantly alleviated.
(2) Under the condition of multi-pore defect combinations, the connection angle between pores is a crucial factor playing a decisive role, whereas the relative spacing of pores has a minor impact. When double pores are arranged horizontally, the strong stress field interference between the pores causes the crack to instantaneously penetrate with extremely low energy consumption, dropping the structural peak load to the global minimum. As the connection angle gradually increases, the stress field interference between the two pores is substantially weakened, and the bearing capacity significantly rebounds and stabilizes. Increasing the connection angle between pores or introducing random inclination angles can effectively induce the deflection of the main crack, thereby enhancing the bearing capacity limit of the overall structure.
(3) This study on the size combination of double pores indicates that there is a critical size threshold for the impact of secondary defects on structural strength. When the secondary pore is a microscopic pore with a horizontal projected length less than 0.1 mm, it is difficult to form effective stress field coupling with the primary pore, and the overall peak load basically maintains a high plateau. However, when the small pore is a mesoscopic pore with a horizontal projected length greater than 0.1 mm, it produces a synergistic failure effect with the primary pore, leading to a significant near-linear decrease in bearing capacity.
(4) The dense aggregation of local defects alters the crack propagation path, making structural failure no longer limited by a single maximum pore. When small pores closely approach each other in space, the intense stress superposition forms a combined defect with a stronger weakening effect. Once its lateral influence range surpasses that of an isolated large pore, the main crack preferentially penetrates the small pores and completely bypasses the large one. Therefore, the local aggregation of small defects is an important factor inducing the abnormal decline in structural bearing capacity and asymmetric failure.

In summary, this study reveals that the interlayer mesoscopic pores in 3DPC not only weaken the material in their independent forms but also trigger multi-crack competitive failure through complex spatial arrangements and stress field interference. This finding indicates that in future optimization of the 3D printing extrusion process, in addition to focusing on reducing the overall porosity, special attention should be paid to suppressing the dense horizontal aggregation and collinear connection of bubbles between layers in order to fundamentally improve the mechanical anisotropy and interlayer weakness of printed structures.

## Figures and Tables

**Table 1:** Calculation parameters of PF_CZM.

Model	$c_{a}$	$a_{1}$	$a_{2}$	$a_{3}$	m	ξ
PF_CZM	π	$\frac{4G_{c}\cdot E_{o}}{\pi l\cdot {f_{t}}^{2}}$	1.3868	0.6567	2.0	2.0

**Table 2:** Calculation parameters of plain concrete.

Parameter	Symbol	Value
Elastic modulus	$E_{0}$ (GPa)	30.0
Poisson’s ratio	v	0.167
Regularization width	*l* (mm)	4.0
Griffith’s constant	*G_c_* (N·mm^−1^)	100
Tensile strength	$f_{t}$ (MPa)	2.5
Compressive strength	$f_{c}$ (MPa)	29.1

## Data Availability

The original contributions presented in the study are included in the article, further inquiries can be directed to the corresponding author.
